# Modelling the impact of sodium intake on cardiovascular disease mortality in Mexico

**DOI:** 10.1186/s12889-023-15827-0

**Published:** 2023-05-26

**Authors:** Jorge Vargas-Meza, Eduardo Augusto Fernandes Nilson, Claudia Nieto, Neha Khandpur, Edgar Denova-Gutiérrez, Isabel Valero-Morales, Simón Barquera, Ismael Campos-Nonato

**Affiliations:** 1 Research Center of Nutrition and Health, National Institute of Public Health, Cuernavaca, Mexico; 2 El Poder del Consumidor A.C., Ciudad de México, Mexico; 3 Center for Epidemiological Research on Nutrition and Health, School of Public Health, University of Sao Paulo, Sao Paulo, São Paulo, Brazil; 4 Programa de Alimentação, Nutrição e Cultura, Oswaldo Cruz Foundation (Fiocruz), Brasília, Brazil; 5 Department of Nutrition, University of São Paulo, São Paulo, Brazil; 6 Department of Nutrition, Harvard T.H. Chan School of Public Health, Boston, USA

**Keywords:** Sodium, Sodium reduction, Cardiovascular disease, Hypertension, Food policy, Public health, Global health

## Abstract

**Background:**

Cardiovascular diseases (CVD) represent the main cause of death in Mexico, while high blood pressure is suffered by about half of the adult population. Sodium intake is one of the main risk factors for these diseases. The Mexican adult population consumes about 3.1 g/day, an amount that exceeds what is recommended by the World Health Organization (WHO) < 2 g sodium/day. The objective of this study was to estimate the impact of reducing sodium intake on CVD mortality in Mexico using a scenario simulation model.

**Methods:**

The Integrated Model of Preventable Risk (PRIME) was used to estimate the number of deaths prevented or postponed (DPP) due to CVD in the Mexican adult population following the following sodium intake reduction scenarios: (a) according to the WHO recommendations; (b) an “optimistic” reduction of 30%; and (c) an “intermediate” reduction of 10%.

**Results:**

The results show that a total of 27,700 CVD deaths could be prevented or postponed for scenario A, 13,900 deaths for scenario B, and 5,800 for scenario C. For all scenarios, the highest percentages of DPP by type of CVD are related to ischemic heart disease, hypertensive disease, and stroke.

**Conclusions:**

The results show that if Mexico considers implementing policies with greater impact to reduce sodium/salt consumption, a significant number of deaths from CVD could be prevented or postponed.

**Supplementary Information:**

The online version contains supplementary material available at 10.1186/s12889-023-15827-0.

## Background

Noncommunicable chronic diseases (NCD) represent the leading cause of death worldwide. Among NCDs, cardiovascular diseases (CVD) are the leading cause of death globally accounting for about 33% of total deaths in 2019 [1]. In the same year, high blood pressure (HBP) was identified as the leading risk factor of death, with a global prevalence of 1,280 million among adults aged between 30 and 79 years [2, 3]. High sodium intake has been identified as among the main risk factors for the development of HBP and CVD, accounting for 9.2% of all CVD deaths worldwide, according to the Global Burden of Disease (GBD) study [3].

These global trends are particularly critical among low and middle-income countries, such as Mexico. In 2019, CVD accounted for 23% of total NCD deaths in Mexico, while HBP was identified as the second leading risk factor of mortality in the country [3], with a prevalence of 50% in the adult population [4]. A diet high in sodium is estimated to account for about 9,000 deaths from cardiovascular causes, of which 14.7% were for hypertensive heart diseases, 10.7% for stroke and 8.5% for ischemic heart disease [3].

NCDs are projected to cost more than US $30 trillion dollars by the end of this decade (2030), which could represent close to 50% of the world’s Gross Domestic Product (GDP) [5]. In Mexico, the treatment of NCDs represents an economic burden of approximately US $2 billion dollars to the federal health system each year [6] and just CVDs alone represent 4% of the total spending on medical care (close to US$ 6.1 million per year) [7].

The World Health Organization (WHO) recommends that sodium intake be less than 2 g per day [8], based on scientific evidence on the direct relationship between sodium intake and the risk of hypertension and CVD [9]. Global mean sodium intake is 3.9 g per day [10], and between 3.4 and 4.1 g per day in Latin America [11]. In Mexico, sodium intake was estimated to be 3.1 g per day among adults, with 64% of the adult population consuming amounts above the WHO recommendations [12].

The reduction of sodium intake at the population level has been suggested as one of the most cost-effective strategies to reduce the risk of CVD´s and premature deaths [13, 14]. Different strategies to reduce sodium consumption – behavior change counseling, food taxes, communication campaigns, front-of-package labeling of foods, and reformulation of industrialized foods - have been implemented in different contexts [15]. In addition, WHO has designed the SHAKE package as a tool for developing countries to design, implement and monitor evidence-based sodium reduction strategies [16]. The SHAKE package is based on “best buy” interventions for reducing population sodium consumption and aims to support countries in achieving the global goal of 30% reduction in sodium intake by 2025 [17].

In Latin America, different countries have implemented strategies to reduce sodium intake. For example, Brazil, Chile, and Argentina have implemented reformulation standards for foods with high sodium content [18–20]. Argentina stands out as the first country in the Americas to develop this as a mandatory regulation [21]. Additionally, Costa Rica and Colombia have developed communication campaigns to increase population awareness of the harmful effects of high sodium consumption on health [22, 23] and several Latin American countries (Chile, Uruguay, Peru, Argentina, Mexico, and Brazil) have implemented front-of-package labels that signal the presence of excessive amounts of sodium in foods products [21, 24–28].

In order to implement public policies or strategies it is necessary to estimate the impact of such interventions on health, including modelling the economic impacts or benefits of the sodium/salt reduction [29–31]. Brazil has estimated that reducing the average intake to the WHO recommendation could prevent 46.6 thousand CVD deaths [29], while Costa Rica estimated that a 15% reduction in sodium intake during 2013 (4 g per day) would prevent 4% of CVD deaths [30]. In addition, in Brazil up to US $102 million dollars could be saved if sodium consumption was cut to 2 g per day [32].

Data obtained through health modeling methodologies can support policy decisions by estimating and comparing the potential impact of health interventions [33]. However, few studies have been conducted on the impact of dietary interventions and policies, as well as evidence on the epidemiological burden of dietary risk factors, such as sodium intake on CVD in Mexico. Given the context of Mexico, it is deemed important to assess the impact of a sodium reduction intervention on health. Therefore, the objective of the present study was to estimate the impact of reducing sodium intake on CVD mortality in Mexico using a comparative risk assessment model for the year 2018.

## Methods

### Simulation model

The Preventable Integrated Risk Model (PRIME) [34, 35] was used to estimate the impact of CVD deaths that would be prevented or postponed in the Mexican adult population by reducing total sodium consumption in the country, complying with the recommendation of the WHO (< 2 g/day).

PRIME is a comparative risk assessment model designed to perform macrosimulation analyses for estimating the impact of different behavioral risk factors (including dietary risk factors such as sodium consumption) on NCD mortality, based on Microsoft Excel [34]. The model uses the changes in the prevalence of the risk factors on the population attributable fraction comparing the baseline data to counterfactual scenarios to estimate the impact on deaths associated to the NCD risk factors, using relative risk from meta-analysis of previously published epidemiological studies. As its main inputs, the model requires sociodemographic data of the population, such as the distribution of the population and deaths for each NCD stratified by age and sex, and the means and standard deviations of the risk factors of interest for the baseline and hypothetical (counterfactual) scenarios.

For the purposes of the model, sodium intake was converted into the equivalent salt intake and specifically for this study, we have only modeled the changes in sodium intake and assumed all other risk factors remained unchanged. PRIME estimations are based on the rationale that changes in sodium intake impact systolic blood pressure, which then impacts the risk of CVD mortality, which are parametrized in the model. Model parametrization involves, firstly, generating a log-linear distribution of salt consumption at baseline and in the counterfactual scenario using the population and the mean salt consumption and its standard deviation to estimate the prevalence of salt consumption for discrete intervals for 5-year age groups for the population over 20 years of age for both sexes. Then, the relative risks of changes in salt intake on blood pressure are linked to the relative risk of changes in blood pressure and for each CVD outcome. Finally, the prevalence of salt intake is used together with the parametrized relative risks to estimate the population attributable fraction (PAF) for changes in salt intake and CVD outcomes for each CVD considering the salt intake intervals for each age and sex group. The PAFs are then used to estimate the attributable deaths from each CVD. The PRIME model is a free and open application available by its developers [34].

### Baseline scenario

Sodium intake was based on a previous study that used information from the National Health and Nutrition Survey 2016 (ENSANUT 2016) [36]. This study estimated mean sodium intake by population groups from 24-hour food recall questionnaires using the Mexican Food Database [36]. Information on CVD related deaths was obtained from the General Management of Health Information (DGIS, by its Spanish acronym) of the Mexican Ministry of Health for the year 2019 [37]. Mortality data were based in the WHO International Classification of Diseases (ICD) [38]. For this study, we considered the following CVDs: ischemic heart disease, stroke, hypertensive disease, heart failure, aortic aneurysm, pulmonary embolism, and rheumatic heart disease. All the data inputs for the model were stratified by sex and age in 5-year intervals starting at 20 years of age (S1 Table 1).

### Counterfactual scenarios

First, we modeled the impact on mortality associated with a per capita intake of less than 2 g of sodium /day for the whole Mexican adult population.

Furthermore, as more realistic mid-term policy scenarios, we proposed two scenarios of sodium reduction: (A) an intermediate scenario where mean sodium intake per day is reduced by 10% of baseline consumption and (B) an optimistic scenario with a 30% reduction in the baseline mean sodium intake, as the WHO goal for 2025 [39] (Table 1). The standard deviations for each counterfactual scenario were estimated assuming that the proportion of the standard deviation to the mean at baseline was maintained in the alternative scenarios.

**Table 1:** Baseline and counterfactual scenarios for sodium intake in Mexico (g/day/person)

	Baseline	Counterfactual Scenarios		
Sex		WHO recommendation	Intermediate Scenario A	Optimistic Scenario B
**Men**	3.4 (1.4)	1.1 (0.4)	3.1 (1.3)	2.4 (1.0)
**Women**	2.9 (0.9)	1.2 (0.4)	2.6 (0.8)	2.0 (0.6)

Data represents mean and standard deviation

WHO, World Health Organization

### Population demographics and health inputs

Demographic information for 2020 was obtained from data of the National Institute of Statistics and Geography (INEGI, by its Spanish acronym) [40]. Diet information (such as energy, fruit, fiber and fat intake), sedentary lifestyle and Body Mass Index (BMI) were obtained from the ENSANUT 2016 [41]. All information was stratified by sex and age and remained unchanged across the counterfactual scenarios for modeling only the effect of changes in sodium intake. The summary of the information sources can be seen in Table 2.

**Table 2:** Summary of the key model inputs and sources for health modeling

Model input	Source
Demographics	INEGI [42]
Salt consumption	ENSANUT 2016 [36]
Deaths from CVD	DGIS [43]
Workforce characteristics	ENSANUT 2016 [44]
Diet	ENSANUT 2016 [44]

### Uncertainty analysis

The uncertainty analysis using Monte Carlo simulation is incorporated into the PRIME model to calculate 95% probabilistic uncertainty intervals (95% UI) for all model outputs, based on 5,000 draws from specified probabilistic distributions constructed on the model input variables. The uncertainty analysis additionally incorporates the usual random error of the relative risks (RR) and sodium intake, to add the other potential sources of uncertainty such as the extrapolation from a source to a target population [45].

## Results

At baseline in Mexico, in 2019, a total of 550,629 deaths occurred due to CVDs (S1 Table 2); 52% of them were among men. If the average sodium intake of the Mexican adult population was less than 2 g/day, as recommended by the WHO, approximately 27,700 deaths from CVDs could be prevented or postponed per year (Table 3).

**Table 3:** Cardiovascular disease deaths prevented or postponed if population sodium consumption was reduced to less than WHO recommendation (2 g/day), Mexico 2019

	Deaths prevented or postponed (95% UI)
**Total**	27,700 (12,489 − 41,822)
**Sex**	
Male	16,700 (7,554 − 25,078)
Female	11,000 (4,959 − 16,726)
**Under 75 years**	14,000 (6,365 − 21,093)
Male	9,600 (4,373 − 14,407)
Female	4,400 (1,996-6,690)
**Cause**	
Ischemic heart disease	13,600 (6,056 − 20,817)
Hypertensive disease	7,800 (3,602 − 11,450)
Stroke	5,200 (2,238-7,887)
Others*	1,100 (457-1,779)

DPP, deaths prevented or postponed

* Others: Heart failure, aortic aneurysm, pulmonary embolism, and rheumatic heart disease

Most deaths prevented or postponed (DPP) from reducing sodium consumption to less than 2 g/day would be among men (60%) and half would be among people under 75 years of age. The major causes of death from CVDs attributable to excessive sodium intake would be ischemic heart disease (49%), hypertensive disease (28%) and stroke (19%).

Table 4 shows estimates of the number of CVD deaths that would be prevented or postponed under the two sodium reduction scenarios for more feasible mid-term goals. Approximately 5,800 CVDs deaths could be prevented under the intermediate scenario (10% reduction), compared to 13,900 under the stricter optimistic (30% reduction).

**Table 4:** Cardiovascular deaths prevented or postponed according to scenarios of sodium intake reduction, Mexico 2019

	Deaths prevented or postponed (95% UI)	
	Intermediate^1^	Optimistic^2^
**Total**	5,800 (2,007-8769)	13,900 (5,915 − 21,807)
**Sex**		
Male	3,100 (1,170-5,443)	7,900 (3,371 − 12,502)
Female	2,100 (839-3,326)	6,000 (2,544-9,307)
**Under 75 years**	2,600 (1,012 − 4,473)	7,000 (2,974 − 10,934)
Male	1,800 (673-3,144)	4,600 (1,942-7,205)
Female	800 (337-1,331)	2,400 (1,032 − 3,736)

^1^ Scenario A: Sodium intake reduction by 10% (3.0 ± 1.2 g/day)

^2^ Scenario B: Sodium intake reduction by 30% (2.4 ± 1.0 g/day) - WHO goal for 2025 [39]

Table 5 shows the deaths that could be prevented or postponed according to the type of CVD for each sodium reduction scenario. According to our estimates, among the total prevented deaths from CVD, most were associated with ischemic heart disease, hypertensive disease, and stroke, for both scenarios. Following, a lower percentage of deaths from other causes would be prevented or postponed, including heart failure, aortic aneurysm, pulmonary embolism, and rheumatic heart disease.

**Table 5:** Cardiovascular deaths prevented or postponed according to scenarios of sodium intake reduction by type of cause1, Mexico 2019

	Intermediate^2^		Optimistic^3^	
	DPP (95% UI)	% DPP	DPP (95% UI)	% DPP
**Total (CVD)**	5,200 (2,007–8,769)	100	13,900 (5,915 − 21,807)	100
Ischemic heart disease	2,400 (936-3,944)	46.2	6,600 (2,811 − 10,388)	47.5
Hypertensive disease	1,600 (616-2,927)	30.8	4,200 (1,756-6,399)	30.2
Stroke	1,000 (371-1,583)	19.2	2,600 (1,092 − 4,064)	18.7
Others *	200 (73–342)	3.8	500 (200–910)	3.6

^1^ The codes within brackets correspond to the International Classification of Diseases codes [46]

^2^ Scenario A: Sodium intake reduction by 10% (3.0 ± 1.2 g/day) for a 2171 kcal/day intake

^3^ Scenario B: Sodium intake reduction by 30% (2.4 ± 1.0 g/day) for a 2171 kcal/day intake- WHO goal for 2025 [39]

DPP, deaths prevented or postponed

* Others: Heart failure, aortic aneurysm, pulmonary embolism, and rheumatic heart disease

## Discussion

This modeling study is the first to show the potential health impacts of reducing sodium intake at the population level in Mexico. This study shows that 15% of deaths attributed to CVDs could be prevented or postponed if dietary sodium intake decreased as recommended by the WHO (< 2 g/day). Among CVDs, a higher percentage of deaths prevented or postponed are observed for ischemic heart disease, hypertensive disease, and stroke regardless of the proposed sodium reduction scenarios.

The results are in line with the other modeling studies conducted in Latin America (Costa Rica and Brazil), also using comparative risk assessment models to estimate the health impact of sodium reduction at the population level. Both studies showed that deaths prevented or postponed from reducing sodium intake are mostly from ischemic heart disease, hypertensive diseases, and stroke [30, 47].

Our study shows that the deaths prevented or postponed would be higher among men if sodium intake was reduced, in all scenarios. This can be partially explained by the fact that Mexican men consume more sodium than women do (3.4 g/day vs. 2.9 g/day), and more of them did not meet WHO’s sodium intake recommendations in comparison with women (68.0% vs. 60.9%) [36]. Additionally, more men die from CVDs compared to women [48, 49].

Modeling studies represent useful *ex* ante policy evaluation tools by simulating changes in risk factors, including sodium consumption, by estimating the burden of disease and the potential impacts of policy options compared to business-as-usual scenarios.

Although this study did not measure the cost-effectiveness of reducing sodium intake, scientific evidence has shown that reducing sodium intake at the population level is one of the most cost-effective strategies to help reduce CVD risk [13, 50]. Despite the fact that direct and indirect policy measures have been implemented in Mexico to reduce sodium consumption in the population (including front-of-package labelling, prohibiting salt shakers at the tables in restaurants, and sodium reduction in bakery products) [51–53], excessive sodium intake continues to represent an important risk factor for CVD in the country. In this regard, our study showed that reducing sodium intake, regardless of the policy scenario, could contribute to significantly reduce CVD mortality in the country.

In Mexico, the main dietary sources of sodium are processed and ultra-processed products, which contribute between 39 and 50% of total sodium consumption [36]. The purchases of these foods have increased in the Mexican population during recent decades [54], therefore strategies to mitigate the problem, such as promoting food reformulation, restricting sales and publicity of high sodium foods and other initiatives should be considered.

Food reformulation for sodium reduction is considered a best buy in terms of cost-effective strategies to prevent cardiovascular disease and many countries have set targets for sodium content in industrialized foods [55, 56]. For example, in the US, a reduction of 25% sodium content in the top 10 food groups that contribute to sodium intake would represent an 8.7% reduction of the total sodium consumption of the adult population [57]. Also, achieving the voluntary sodium targets proposed by the Food and Drug Administration could prevent or postpone 13 to 35 thousand deaths from CVD in US over the 20-year simulation period [58].

Additionally, in Brazil the voluntary strategy to gradually reduce the sodium content in industrialized foods achieved an 8 to 34% reduction of the sodium content in target products 6 years after the implementation of the first sodium targets, although this contributed to a modest 0.1 g/day Reduction in the average sodium intake in the country [59, 60]. Even though, this voluntary reduction strategy was estimated to prevent or postpone approximately 110 thousand CVD cases and 2,600 deaths from CVD over a 20-year period [61]. However, the adoption of regulatory measures and more stringent targets could prevent more than threefold the impact of actual food reformulation [62, 63]. Similarly, a recent study modeling the dietary and health impact of full compliance with WHO sodium reference values in Australia showed that it could reduce the average sodium intake of adults by 404 mg/day, corresponding to a 12% reduction. This reduction showed that it could prevent about 1,770 deaths/year (UI95%: 1,168-2,587), corresponding to 3% of all deaths from cardiovascular disease, chronic kidney disease and stomach cancer in Australia, and prevent about 6,900 (UI95%: 4,603-9,513) new cases, and 25,700 (UI95%: 17,655 − 35,796) disability-adjusted life years/year in the country [64].

Likewise, Argentina firstly set voluntary sodium targets for key food categories, but later transitioned to mandatory targets, with stronger enforcement mechanisms, and, as a result, more than 90% of the food products met the established goals for sodium reduction within by year 2018 [21, 65].

It is therefore likely that implementing food reformulation targets could help reduce sodium consumption in Mexico so that sodium intake is reduced to levels close to the intermediate scenario of this study and thereby produce positive health outcomes for the population.

Based on the existing sodium reduction targets in many countries, the WHO has proposed a set of global benchmarks for food groups that contribute the most to sodium intake [55]. Similarly, the Pan American Health Organization (PAHO) has updated and expanded its regional sodium targets, which are important references for planning and implementing national food reformulation targets [56].

Recently in Mexico, a front-of-package nutritional labeling system has been implemented through a warning system based on the Chilean design [66] and the PAHO nutritional profile for nutrient thresholds [67]. Because this regulation is recent, there is no available evaluation of the results of this policy yet; however, it has been observed that, before entering into force, different food companies already reformulated their foods by reducing the sodium content [68, 69], and popular brands developed reduced- or low-sodium options for the Mexican market [70]. Likewise, in Chile, the evaluation of labeling policy proposed that the warning labels had frequently induced the reformulation of high-sodium products [71]. However, despite the implementation of front-of-package labels in Mexico, it has been proven that reducing sodium consumption in the country requires multiple component strategies to produce greater impacts and achieve its main policy objectives [15, 72].

Based on the results of this study, it is important that decision makers consider the implementation of a set of sodium reduction policies in Mexico. For example, countries that have implemented multiple coordinated strategies to reduce sodium consumption, such as the United Kingdom and Finland, have shown impacts on health outcomes, particularly on reducing blood pressure at the population level and reducing the burden of CVD, such as ischemic diseases and cerebrovascular accidents, and even improving life expectancy in 5 to 6 years [73–75].

This study has some strengths and limitations. The strengths of this study include the use of nationally representative data from administrative records and national surveys. Also, the comparative risk assessment methodologies estimations are based on country-specific data and robust meta-analysis for the relative risks of dietary risk factors, such as sodium. These macrosimulation approaches provide estimations that allow inter-country and regional comparisons and can be applied to other years or periods of years from data commonly available national and international databases.

The main limitations of the study are as follows. Sodium intake was estimated indirectly from 24-hour food recalls in a national survey, which likely underestimates sodium consumption at the population level and, furthermore, it was not designed to identify total sodium intake [76]. For example, only salt in preparation was measured and not added table salt). These limitations may result in underestimation of deaths prevented or postponed (DPP) Future studies could consider measuring sodium intake through a more accurate method, like 24-hour urine collection, in order to provide more accurate inputs for epidemiological and economic modeling. Additionally, the modeled estimates in this study were based on sodium intake data from the ENSANUT 2016 due to the lack of information published in more recent surveys. This may have lowered the DPP estimates when contrasting them with the mortality information from more recent official records (2019). However, according to GBD data, the percentage of total CVD deaths was similar between the years 2016 and 2019 (22.63% vs. 22.69%, respectively) [3]. In addition, despite using t the most recent nationally representative data available in the country on sodium, sodium intake of Mexican adults may have changed between 2016 and 2019.

Another limitation of this study is that the PRIME model is based on a cross-sectional approach and does not consider longitudinal analyses and lag-times between changes in risk exposures and health outcomes. However, the model has been used by several studies in different countries [77] because of its adaptability to different settings and its capability of estimating the direct impact of dietary changes on mortality and incorporating the uncertainty of data inputs through Monte Carlo simulations.

Finally, the counterfactual scenarios have failed to include the effects of several important dietary sources of sodium despite. Of particular importance most of the sodium in the diets of Mexicans comes from processed and ultra-processed foods. Therefore, an appropriate design of future studies is to focus on the health effect of simulated interventions in the reduction of sodium consumption in these food groups in Mexico and on more detailed analyses that consider age and sex differences in the population.

## Conclusions

Our findings show that regardless of the setting, a large number of deaths could be prevented if reductions in sodium intake were achieved. Therefore, it is important to promote interventions to reduce the consumption of this nutrient, since CVDs are largely preventable. International organizations suggest the implementation of multiple components (mandatory food reformulation, front-of-pack labeling, nutritional advice, and media campaigns) to achieve more significant impacts on population-level sodium intake and CVD outcomes. Mexico could consider the implementation of comprehensive strategies that involve different components to reduce the sodium intake of the population.

## Electronic supplementary material

Below is the link to the electronic supplementary material.

Supplementary Material 1

## Data Availability

All relevant input data are within the paper and in the supporting information files and were obtained from publicly available national databases from Mexico. Demographic information is available from the National Institute of Statistics and Geography – INEGI (https://www.inegi.org.mx/temas/estructura/), mortality data is available from the General Directorate of Health Information – DGIS (http://www.dgis.salud.gob.mx/contenidos/basesdedatos/bdc_defunciones_gobmx.html) and diet and workforce characteristics are available from the National Health and Nutrition Survey – ENSANUT 2016 (https://ensanut.insp.mx/encuestas/ensanut2016/index.php) The PRIME Model is available by request to its developers.
