# Supplementary material for: Modelling the impact of sodium intake on cardiovascular disease mortality in Mexico
Source: BMC Public Health. 2023 May 26;23:983. doi: 10.1186/s12889-023-15827-0 (PMC10224594; doi:10.1186/s12889-023-15827-0)
Supplement: Supplementary file 1 — Supplementary Material 1 [file 12889_2023_15827_MOESM1_ESM.pdf]

Supplementary materials

Modelling the impact of sodium intake on cardiovascular disease mortality in Mexico

Jorge Vargas-Meza, Eduardo Augusto Fernandes Nilson, Claudia Nieto, Neha Khandpur, Edgar Denova-Gutiérrez, Isabel Valero-Morales, Simón Barquera, Ismael Campos-Nonato

BMC Public Health

Materials and methods

CVD MORTALITY MODULE

Modeling of prevented or postponed deaths

PRIME model and estimation of the effect of reduction of salt intake on the mortality from cardiovascular diseases.

The Preventable Risk Integrated ModEl (PRIME) is a macro-simulation model which was developed to estimate the impact of changes in the distribution of NCD risk and protection factors, including dietary variables, on mortality from chronic diseases. The complete PRIME model uses inputs such as dietary consumption (including alcohol consumption) and nutrient intakes, physical activity, height, BMI, and smoking status.

Mortality and demographic data

The cardiovascular diseases related to excessive salt intake used in the model include coronary heart disease, stroke, hypertensive disease, heart failure, aortic aneurysm, pulmonary embolism, and rheumatic heart disease (ICD-10: I05-15, I20-26, I50, I60-69, I71). Mortality data for each disease was obtained from publicly available databases of the, stratified by sex and five-year age groups and ICD-10 codes.

Baseline and counterfactual scenario of dietary distribution

The dietary intakes of salt from the Sodium intake was based on a previous study that used information from the National Health and Nutrition Survey 2016 (ENSANUT 2016). This study estimated mean sodium intake by population groups from 24-hour food recall questionnaires using the Mexican Food Database. Information on CVD related deaths was obtained from the General Management of Health Information (DGIS, by its Spanish acronym) of the Mexican Ministry of Health for the year 2019. Mortality data were based in the WHO International Classification of Diseases (ICD). For this study, we considered the following CVDs: ischemic heart disease, stroke, hypertensive disease, heart failure, aortic aneurysm, pulmonary embolism, and rheumatic heart disease. All the data inputs for the model were stratified by sex and age in 5-year intervals starting at 20 years of age (S1 Table1). We assumed that the standard deviation (SD) of salt intake in the counterfactual scenario was proportional to the SD at the baseline.

For estimating the sodium intake in each counterfactual scenario, we assumed that the new standard deviations remained at the same proportion to the mean consumption as that observed at the baseline. Additionally, for estimating the mean intake and the standard deviation for the WHO recommendations (sodium consumption of less than 2g/day), we considered that, in a normal distribution of sodium intake in the population, so that over 97.5% of the population would consume than 2g of sodium per day by applying the formula: Mean intake ± 2 x SD < 2 g.

Parameterization of the association between dietary consumptions and chronic diseases

The PRIME model estimates death rates associated to chronic diseases in a given situation using relative risks of different levels of nutrient intakes on chronic diseases and the baseline distribution. In the case of salt intake, the model has a two-step approach: first, it simulates the impact of changes in salt intake on blood pressure, and then it simulates the impact of the changes in blood pressure on the number of deaths averted or delayed from cardiovascular diseases. The model uses a log-normal distribution of salt intake in the population, for both the baseline and counterfactual scenarios, for sex and 5-year age groups, using the mean and standard deviation of salt intake and the population data. The net impact of the changes in the risk factor is represented by the difference of the averted deaths number between the baseline and counterfactual scenarios.

Uncertainty analyses

Considering the uncertainty of outcomes in the model, performing a probabilistic sensitivity analysis is recommended in order to explore the potential effects of reducing salt consumption on the risk factors for CVDs. In this paper, simulations

were performed using the Monte Carlo methodology, which allows a stochastic (random) variation of parameters based on the sizes of the effects obtained from the literature. By using this technique, the model results were recalculated iteratively and uncertainty intervals of 95% (UI 95%) were generated for the median using the bootstrap percentile method. The model simulation was implemented the Monte Carlo analysis embedded in the PRIME and running 10,000 iterations (draws) from specified probabilistic distributions for the model input variables (salt intake, deaths and relative risks).

The macrosimulation models (both PRIME and cost evaluation) implement a Monte Carlo approach to estimate uncertainty intervals (UI) for each scenario. Each simulation runs 10,000 times. For each iteration, log-normal distributions of salt consumption, together with the relative risks from literature, are assumed for the input parameters.

The macrosimulation framework does not allow stochastic uncertainty, such as microsimulations (patient-level models), nevertheless parameter uncertainty and individual heterogeneity in our study are reflected in the reported UI. Modeling patient heterogeneity allows analyses based on individual patient characteristics that can influence the outcomes of a decision model. In this study, we have modelled discrete subgroups to represent patient heterogeneity, considering gender or age ranges, within which all individuals are assumed identical. The subgroup-specific characteristics result in subgroup-specific expected outcomes and the discrete distribution of the expected outcome across all subgroups reflects the patient heterogeneity. In addition, parameter uncertainty (2nd order uncertainty) expresses the results from lack of perfect knowledge on their true values, so parameters as relative risks are typically represented by a probability distribution that can be propagated through the model using Monte Carlo simulation, resulting in a distribution of the expected outcome, reflecting lack of perfect knowledge.

Therefore, we allow the risk for CHD to be conditional on individual characteristics (i.e. age, sex, exposure to risk factors – sodium intake) and consider the estimate the uncertainty of the relative risks due to sampling errors through the Monte Carlo analyses.

The structure of the models is grounded on fundamental epidemiological ideas and well-established causal pathways; therefore, we considered this type of uncertainty relatively small and did not study it.

Parameter estimation and uncertainty follow the Modeling Good Research Practices, by incorporating different concepts related to uncertainty, including the stochastic (first-order) uncertainty, the parameter (second-order) uncertainty, the structural uncertainty and the heterogeneity.

The framework of these macrosimulations allows stochastic uncertainty, parameter uncertainty, and individual heterogeneity to be reflected in the reported UI. In this

kind of modelling, the heterogeneity encompasses the variability between patients that can be attributed to their characteristics, which in regression terms, would correspond to beta coefficients or the extent to which dependent variable varies by patient characteristics. In the case of these models, which are based on the sodium-hypertension-cardiovascular outcome rationale, the four sources of uncertainty are incorporated in the model, through Monte Carlo analysis, parameter parametrization and assumptions of the decision model (as the log-linear regression for the distribution of salt consumption). As macrosimulations, the heterogeneity is not considered at the individual level (as for microsimulations) but is assessed though the different exposures (sodium intake) and the parametrized relative risks that are specific to each exposure level, age-group and sex, in order to allow the reproducibility of the results.

The uncertainties, therefor, were incorporated in the final UI by implementing a 2nd order Monte Carlo analysis to estimate uncertainty in each scenario. The 2nd order Monte Carlo analysis uses two loops of iterations: the inner loop represents the variability (as the SD of the exposures) and the outer loop represents parameter uncertainty (as the RRs used in the parametrization). This also allows the model to incorporate the usual random error (sampling error) in the RR and exposure prevalence as well as other potential sources of uncertainty such as uncontrolled confounding or extrapolation from a source to a target population, because of the assumption of the portability of the RRs from the metanalyses. In case of the modeling uses in this study, the final potential impact fractions (PIF) are based on the weighted sum of the PIF for each exposure, sex and age-group strata. Again, after repeated draws and repeated calculations of the PIF, Monte Carlo limits can be obtained. Patient heterogeneity is represented by frequency distributions and analyzed with Monte Carlo simulation. Parameter uncertainty is represented by probability distributions and analyzed with 2nd-order Monte Carlo simulation (aka probabilistic sensitivity analysis).

Supplementary tables

Table S1. Mexican population data according to age group and sex, 2020.

Age (years)	Male	Female
20-24	5,165,884	5,256,211
25-29	4,861,404	5,131,597
30-34	4,527,726	4,893,101
35-39	4,331,530	4,688,764
40-44	4,062,304	4,441,282
45-49	3,812,344	4,130,069
50-54	3,332,163	3,705,369
55-59	2,692,976	3,002,982
60-64	2,257,862	2,563,200
65-69	1,706,850	1,938,227
70-74	1,233,492	1,413,848
75-79	847,898	966,684
80-84	523,812	651,552
85+	433,968	605,593

Source: National Institute of Statistics and Geography.
Available at: https://www.inegi.org.mx/temas/estructura/#Tabulados

Table S2. Total deaths from CVD in the Mexican population according to age group and sex, 2019.

Male age (Years)	Cerebrovascular deseases	Ischemic heart diseases	Lip, oral cavity and pharynx	Oesophagus	Stomach	Bronchus and lung	Pancreas	Colorectum	Breast	Endometrium	Gallbladder
15-19	54	130	1	0	2	5	0	4	0	0	1
20-24	87	286	3	1	21	17	4	12	0	0	0
25-29	115	448	9	4	30	18	8	27	0	0	0
30-34	193	679	10	6	65	23	20	61	0	0	1
35-39	287	968	12	12	95	24	24	88	0	0	0
40-44	437	1,554	27	26	138	80	50	145	2	0	1
45-49	643	2,281	30	32	174	89	104	221	0	0	1
50-54	920	3,215	53	61	242	165	148	299	5	0	15
55-59	1,141	4,269	91	87	303	353	215	401	4	0	15
60-64	1,419	5,423	114	117	393	447	319	469	4	0	20
65-69	1,715	6,004	132	127	421	567	364	482	7	0	31
70-74	2,048	7,050	115	131	456	666	313	487	7	0	25
75-79	2,368	7,547	100	102	436	661	283	424	2	0	27
80-84	2,474	8,130	81	83	311	541	204	343	4	0	22
M85+	3,603	15,202	75	70	300	414	152	291	13	0	13
**Total**	**17,504**	**63,186**	**853**	**859**	**3,387**	**4,070**	**2,208**	**3,754**	**48**	**0**	**172**
**Female age (years)**											
15-19	49	59	1	0	4	4	0	4	3	1	0
20-24	49	69	5	0	8	5	3	10	8	0	1
25-29	71	124	3	1	34	6	6	22	40	1	2
30-34	104	164	9	2	63	14	13	41	153	8	1
35-39	169	303	10	6	70	30	16	89	332	15	6

40-44	286	505	17	9	139	56	45	132	535	31	7
45-49	435	747	22	9	192	112	92	179	799	60	33
50-54	593	1,284	28	10	233	173	156	283	924	68	53
55-59	730	1,777	38	20	285	238	218	299	982	124	53
60-64	1,129	2,594	44	25	314	304	293	351	922	171	76
65-69	1,362	3,479	55	37	349	344	328	431	736	146	76
70-74	1,792	4,568	52	28	348	381	332	362	598	109	78
75-79	2,334	5,891	61	40	318	375	340	337	505	68	61
80-84	2,903	7,812	61	35	265	307	272	289	395	46	53
85+	5,569	20,781	75	35	315	289	234	301	439	36	51
**Total**	**17,575**	**50,157**	**481**	**257**	**2,937**	**2,638**	**2,348**	**3,130**	**7,371**	**884**	**551**

Source: General Directorate of Information. Minister of Health. Government of Mexico.
Available at: http://www.dgis.salud.gob.mx/contenidos/basesdedatos/bdc_defunciones_gobmx.html

(continued) **Table S2.** Total deaths from CVD in the Mexican population according to age group and sex, 2019.

**Male age (years)**	**Kidney**	**Hypertensive disease**	**Diabetes**	**Bladder cancer**	**Liver cancer**	**Cervix cancer**	**Chronic obstructive pulmonary disease**	**Liver disease**	**Heart failure**	**Aortic aneurysm**	**Pulmonary embolism**	**Rheumatic heart disease**	**Chronic renal failure**	**Total**
15-19	1	17	13	0	5	0	12	28	9	3	9	1	62	357
20-24	3	63	47	2	14	0	16	125	20	3	10	2	171	907
25-29	5	126	126	2	13	0	17	366	28	7	17	1	254	1,621
30-34	10	141	270	3	16	0	27	806	32	2	31	5	205	2,606
35-39	13	140	646	5	33	0	41	1520	35	7	36	9	184	4,179
40-44	63	257	893	18	61	0	68	2389	58	12	48	1	194	6,522
45-49	106	374	2461	18	116	0	104	3053	87	13	53	6	322	10,288
50-54	190	486	3958	25	183	0	193	3440	126	16	77	15	388	14,220
55-59	253	671	5725	46	275	0	366	3586	122	26	72	14	504	18,539
60-64	248	849	6587	90	468	0	737	3509	199	36	85	15	571	22,119
65-69	209	982	6659	96	521	0	1019	2781	218	48	87	28	586	23,084
70-74	238	1246	6466	112	517	0	1636	2214	271	69	75	20	608	24,770
75-79	216	1344	5847	141	496	0	2197	1694	324	78	78	20	640	25,025
80-84	130	1644	4628	133	363	0	2391	1109	387	54	62	18	626	23,738
85+	77	2864	4449	134	310	0	4827	876	882	41	111	10	798	35,512
**Total**	**1,762**	**11,204**	**48,775**	**825**	**3,391**	**0**	**13,651**	**27,496**	**2,798**	**415**	**851**	**165**	**6,113**	**287,322**
**Female Age (years)**														
15-19	3	16	19	0	7	1	3	22	10	0	5	1	63	275
20-24	2	43	41	4	7	6	3	26	7	2	12	0	77	388
25-29	6	67	78	2	9	84	3	55	16	3	13	0	133	779
30-34	6	83	186	2	13	163	10	93	12	1	9	3	144	1,297

35-39	14	99	377	3	28	271	17	196	14	2	31	5	124	2,227
40-44	34	166	893	4	52	320	41	288	36	6	33	9	162	3,806
45-49	65	264	1806	10	140	418	77	472	52	7	39	44	223	6,297
50-54	93	362	2883	10	153	431	120	669	78	14	47	31	275	8,971
55-59	112	512	4548	26	289	445	274	1092	121	9	58	43	317	12,610
60-64	149	760	5895	27	386	395	455	1295	166	19	73	49	451	16,343
65-69	140	974	6645	32	446	358	819	1448	191	14	67	49	511	19,037
70-74	112	1238	6697	44	509	341	1205	1356	253	24	86	66	444	21,023
75-79	135	1602	6586	50	491	273	1595	1131	305	34	105	50	452	23,139
80-84	56	2187	6100	57	384	219	2701	815	459	30	91	42	464	26,043
85+	85	5033	7261	79	370	214	5273	644	1342	34	201	36	727	49,424
**Total**	**1,012**	**13,406**	**50,015**	**350**	**3,284**	**3,939**	**12,596**	**9,602**	**3,062**	**199**	**870**	**428**	**4,567**	**263,307**

Source: General Directorate of Information. Minister of Health. Government of Mexico.
Available at: http://www.dgis.salud.gob.mx/contenidos/basesdedatos/bdc_defunciones_gobmx.html
